# The High-Amylose Maize Line GEMS-0067 Is Associated with an Intron 8 Insertion in *Sbe1a*, Altered Native PAGE Zymogram Patterns, and Development of PCR Markers for Breeding Applications

**DOI:** 10.3390/plants15203157

**Published:** 2026-10-15

**Authors:** Mark R. Campbell, Avinash Karn, Marvin Paul Scott, Luke A. Farno

**Affiliations:** 1 Department of Agricultural Research and Development Program, Central State University, Wilberforce, OH 45384, USA; 2 Syngenta Seeds, DeKalb, IL 60150, USA; 3 Department of Agronomy, Iowa State University, Ames, IA 50011, USA; sqlpaulscott@gmail.com; 4 United States Department of Agriculture-Agricultural Research Service (USDA-ARS), Iowa State University, Ames, IA 50011, USA

**Keywords:** maize, amylose, starch branching enzyme 1

## Abstract

The high-amylose maize (*Zea mays* L.) germplasm line GEMS-0067 carries the recessive amylose-extender (*ae*/*sbe2b*) allele together with modifier loci that elevate starch amylose content from approximately 55% to more than 70%. Previous quantitative trait locus (QTL) studies associated a major component of this modifier effect with the *Sbe1a* region on chromosome 5S; however, the underlying DNA polymorphism and practical breeding markers remained unresolved. To identify candidate variation, a targeted genomic amplicon-scanning strategy was applied across coding and non-coding regions of *Sbe1a*. Screening with 23 gene-specific primer pairs identified a unique fragment-length polymorphism spanning the intron 8-exon 9 boundary. Sequencing revealed a 6 bp TACATA insertion together with a downstream 2 bp GA insertion within intron 8. The polymorphism was present in GEMS-0067 and its ancestral source Guatemala 209 but absent from 33 surveyed USDA Germplasm Enhancement of Maize (GEM) releases. Native PAGE zymogram analysis identified a distinct starch-metabolizing enzyme activity band in the putative SBEI region that displayed altered migration in GEMS-0067 relative to reference genotypes. A PCR-CAPS marker based on the insertion was developed using MslI digestion and enabled clear codominant discrimination of homozygous and heterozygous individuals. These results establish a novel gene-based marker associated with the GEMS-0067 high-amylose modifier background and provide a practical tool for marker-assisted breeding.

## 1. Introduction

Maize (*Zea mays* L.) starch with elevated levels of amylose—typically referred to as high-amylose starch or amylomaize—represents an invaluable raw material for both industrial applications and nutritional science. High-amylose starches exhibit unique functional properties, including elevated gelatinization temperatures, increased film-forming capabilities, and resistance to enzymatic digestion. This last property leads to the formation of resistant starch, a dietary fiber component with proven benefits for human gastrointestinal health and glycemic regulation [1]. Consequently, decoding the genetic architecture of starch biosynthesis to systematically drive amylose accumulation is a primary focus of contemporary maize breeding programs.

The primary genetic driver of high-amylose phenotypes in maize is the classical amylose-extender (*ae*) mutant allele. This recessive mutation occurs within the gene encoding starch branching enzyme IIb (*Sbe2b*), which is responsible for introducing α-(1,6) branch points into the growing amylopectin polymer. In standard elite field corn lines, wild-type starch typically comprises approximately 25% amylose and 75% amylopectin. Homozygous inactivation of the *Sbe2b* locus via the *ae* mutation shifts this balance, reducing branching frequency and raising apparent amylose content. The *ae* mutation also alters starch granule morphology, gelatinization characteristics, thermal behavior, and starch chain-length distribution, illustrating the broad influence of SBEIIb activity on endosperm starch structure and functionality [2].

The unique maize germplasm line GEMS-0067 (Reg. No. GP-550, PI 643420) represents an invaluable genetic resource for evaluating these modifier networks. Developed through the public–private USDA Germplasm Enhancement of Maize (GEM) program, GEMS-0067 combines the core recessive *ae* mutant background with tropical allelic diversity derived from the exotic accession Guatemala 209 (PI 498583) [3,4]. This specific genetic combination routinely yields a stable phenotype exceeding 70% amylose. Subsequent quantitative trait locus (QTL) mapping studies using an endosperm-based inheritance framework identified a major genomic region on the short arm of chromosome 5 (5S) that explains a substantial proportion of this elevated amylose variation [5,6]. Because this specific interval contains the starch branching enzyme 1a (*Sbe1a*) gene, *Sbe1a* became the primary candidate locus associated with the modifier effect.

Subsequent molecular investigations successfully identified sequence polymorphisms within the coding regions of *Sbe1a*, confirming its tight linkage to the high-amylose phenotype and enabling the development of molecular markers associated with the high-amylose phenotype [7,8].

While these marker systems established *Sbe1a* as a premier modifier locus, they relied almost exclusively on Single Nucleotide Polymorphisms (SNPs) and structural variants within exonic regions. Consequently, the potentially significant sequence variation within introns and other non-coding regulatory sequences remains largely unexplored. Identifying these non-coding variants is critical, as intronic sequence variation can profoundly dictate pre-mRNA processing, splice site recognition, and overall translational efficiency in plants. Furthermore, previous marker configurations frequently suffered from low electrophoretic resolution or required labor-intensive methodologies, limiting their practical implementation in high-throughput marker-assisted selection (MAS).

To address these limitations, the present study employed a targeted genomic amplicon-scanning approach across both coding and non-coding regions of the *Sbe1a* locus. The specific objectives were to:

1. Identify DNA polymorphisms within *Sbe1a* that distinguish the high-amylose maize line GEMS-0067 and its derived breeding materials from other GEM germplasms.
2. Determine whether candidate polymorphisms are associated with altered native PAGE zymogram patterns in developing endosperm.
3. Develop and validate a robust codominant PCR-CAPS marker system suitable for high-throughput marker-assisted selection in breeding populations.

Using this approach, we identified a previously undescribed intron 8 polymorphism associated with the GEMS-0067 modifier background and developed a codominant PCR-CAPS marker suitable for marker-assisted breeding applications. Native PAGE zymogram analysis further revealed a distinct enzyme activity-band migration pattern in the putative SBEI region associated with the GEMS-0067 genotype. The biological basis of this association remains unresolved and warrants further investigation.

## 2. Results

### 2.1. Initial Polymorphism Screening Across the Sbe1a Locus

**Figure 1:**
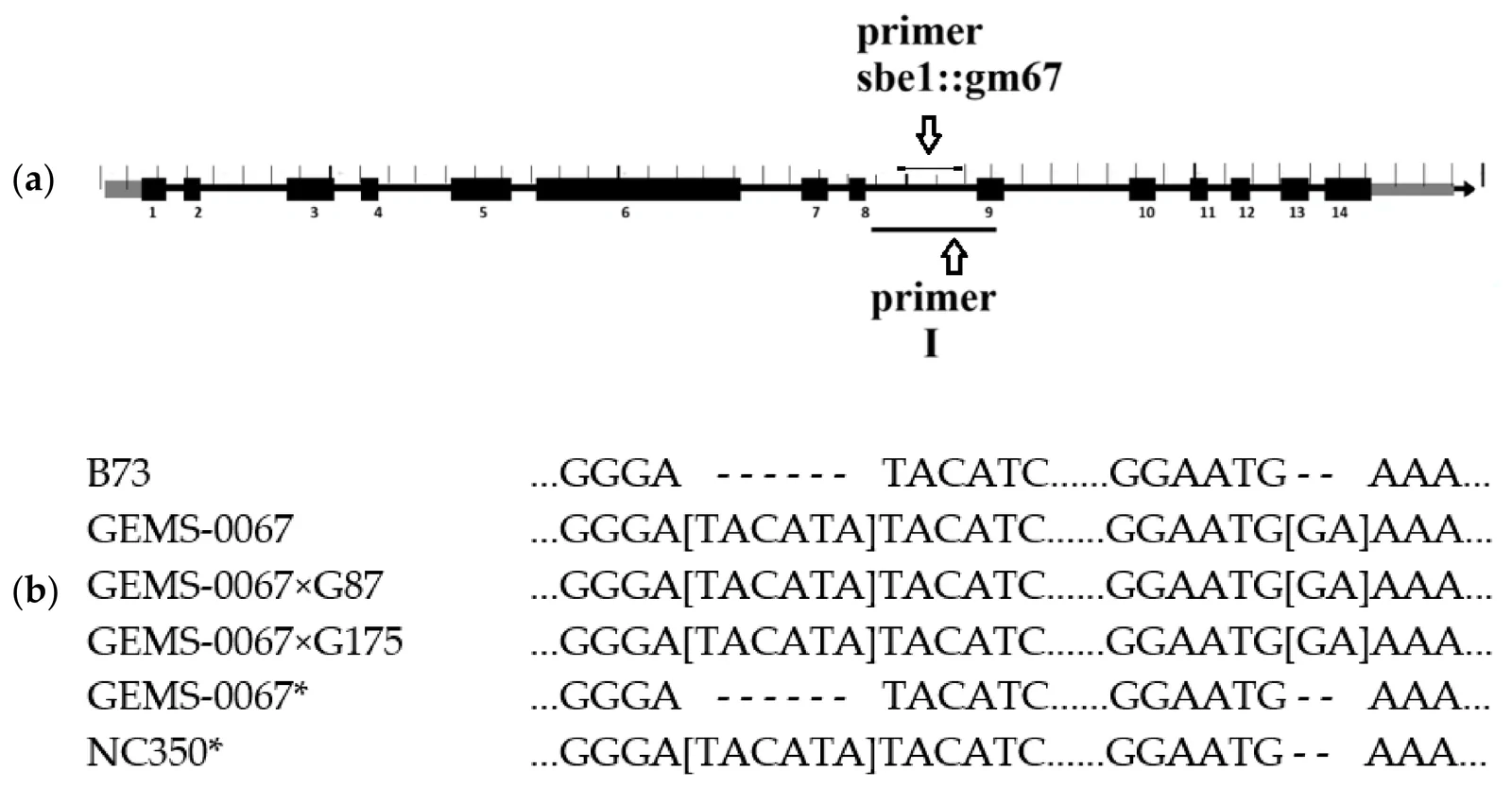
Structural organization and sequence architecture of the *Sbe1a* locus and the *sbe1a:gm67* allele. (**a**) Schematic representation of the *Sbe1a* gene model remapped to the Zm-B73-REFERENCE-NAM-5.0 genome assembly. Black blocks indicate exons (numbered 1–14). Primer I spans the intron 8–exon 9 junction and targets the region containing the diagnostic polymorphism associated with the *sbe1a:gm67* allele. The redesigned *sbe1a:gm67* marker employs a shortened reverse-primer layout to minimize amplicon size while retaining the diagnostic intron 8 region. (**b**) Multiple sequence alignment generated from Sanger sequencing reads of B73, GEMS-0067, and derived high-amylose breeding lines. The diagnostic 6 bp TACATA insertion and downstream 2 bp GA insertion are indicated in brackets. Dashes indicate absence of the insertion relative to the GEMS-0067 allele, and ellipses indicate identical flanking sequence omitted for clarity. Sequences marked with an asterisk (*) were derived from the published data of Gyawali [9] and are shown for comparison with the present study.

**Figure 2:**
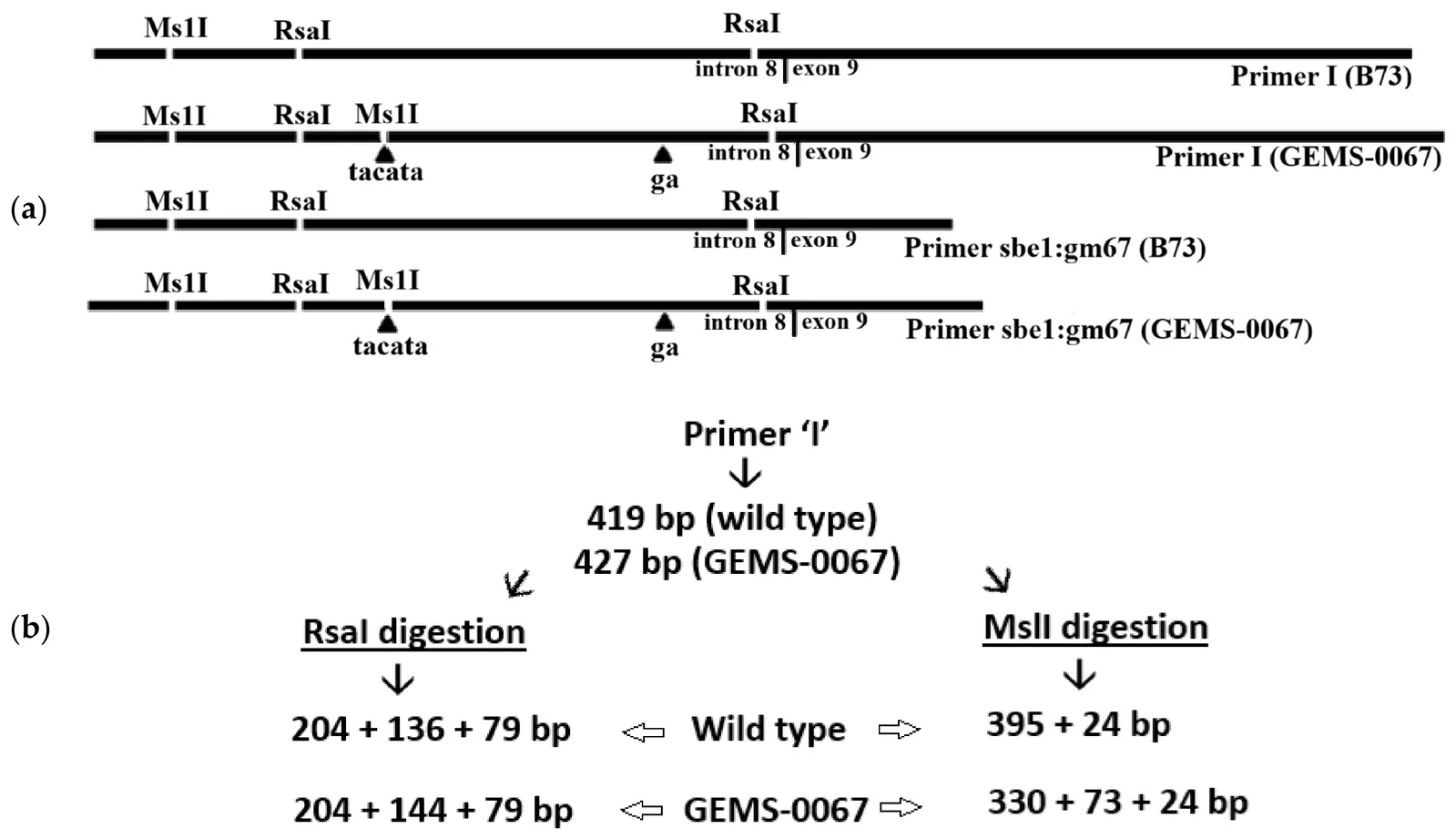
Restriction maps and predicted digestion profiles for Primer I and *sbe1a:gm67* marker assays. (**a**) Restriction maps for the intact Primer I and shortened *sbe1a:gm67* amplicons displaying relative cutting sites for RsaI and MslI endonucleases for B73 and GEMS-0067 alleles. Arrowheads indicate the positions of the 6 bp TACATA insertion and downstream 2 bp GA insertion adjacent to the intron 8–exon 9 boundary. The TACATA insertion creates a novel MslI recognition motif (CATATACATG) unique to the *sbe1a:gm67* allele. (**b**) Comparative schematic diagram showing expected restriction fragment lengths (bp) generated by the codominant RsaI and MslI CAPS marker assays.

**Figure 3:**
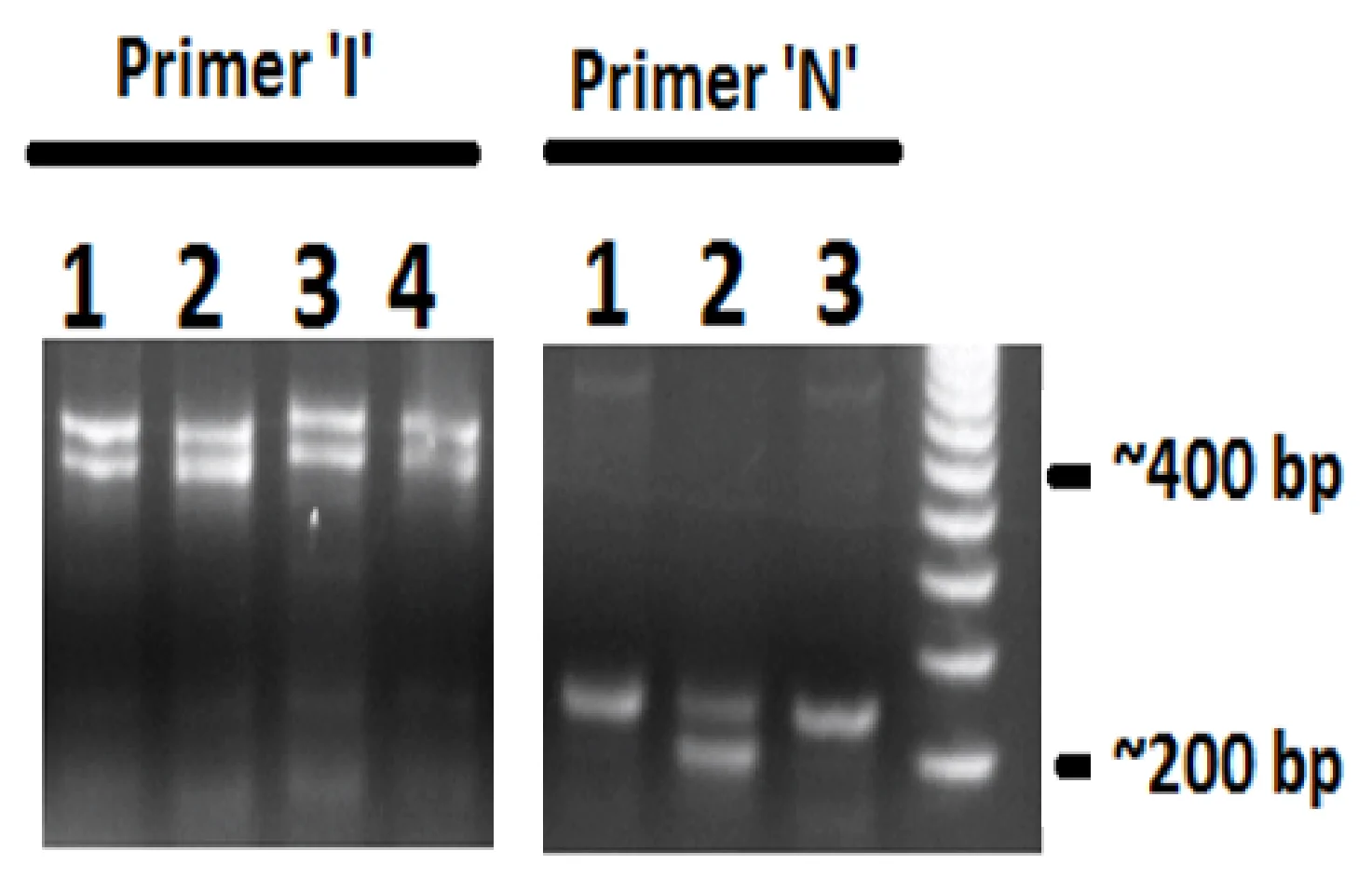
Initial fragment scanning of candidate *Sbe1a* markers. Agarose gel electrophoresis (3.5% agarose, 2 h run) showing amplicon profiles generated by selected candidate primer pairs. Lanes correspond to: 1, Guatemala 209; 2, H99*ae*; 3, GEMS-0067; and 4, GEMN-0087 (not shown for Primer N). Primer I resolved a distinct fragment-length polymorphism, producing an approximately 427 bp amplicon in GEMS-0067 and Guatemala 209 compared with an approximately 419 bp amplicon in H99*ae* and GEMN-0087. Primer N produced additional polymorphic bands; however, these bands did not consistently track with the high-amylose modifier phenotype in subsequent analyses.

A panel of 23 gene-specific primer pairs (A–V) spanning the *Sbe1a* genomic region was evaluated across four control genotypes: H99*ae*, GEMS-0067, Guatemala 209, and GEMN-0087 (Figure 1a, Supplemental Table S2). Out of the 23 tested markers, 21 generated completely monomorphic amplification patterns across all genotypes. Two primer pairs, Primer I and Primer N, produced fragment-length polymorphisms. Primer N amplified a product of approximately 224 bp; however, its alternative banding pattern was not consistently associated with the elevated amylose trait across segregating lines. Primer I, which spans the intron 8–exon 9 boundary region, amplified a fragment-length polymorphism that distinguished GEMS-0067 and Guatemala 209 from H99ae and GEMN-0087. Sequence analysis revealed that the polymorphism consisted of a 6 bp TACATA insertion together with a downstream 2 bp GA insertion located within intron 8 (Figure 1b). The insertion generated a novel MslI recognition site that formed the basis of subsequent CAPS marker development (Figure 2). Electrophoretic analysis revealed the polymorphism b a 419 bp amplicon in H99*ae* and GEMN-0087 and a larger 427 bp amplicon in GEMS-0067 and Guatemala 209 (Figure 3).

### 2.2. Distribution of the sbe1a:gm67 Allele Within USDA-GEM Releases

**Figure 4:**
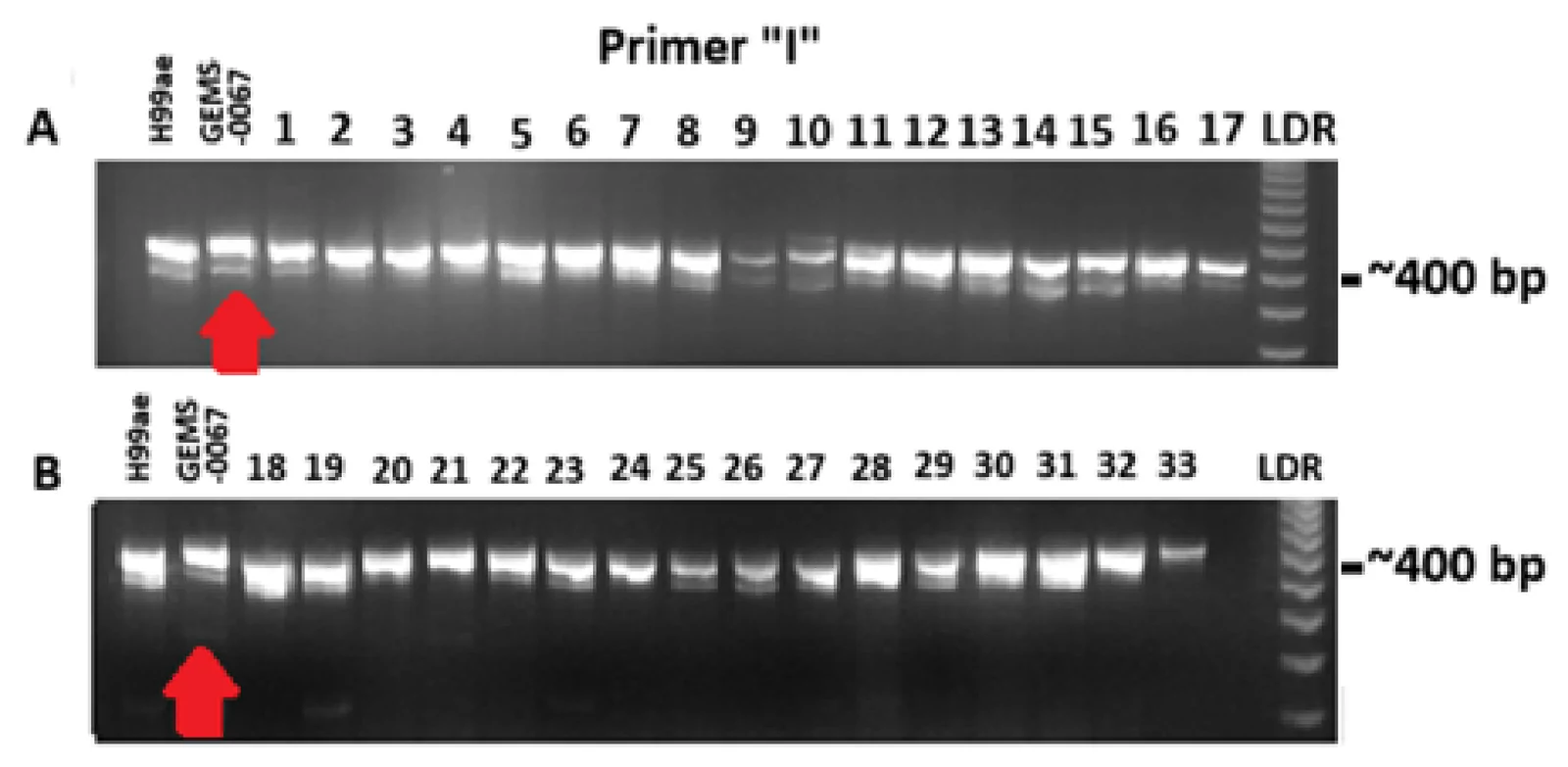
Large-scale allele frequency screening of Primer I across elite USDA Germplasm Enhancement of Maize (GEM) releases. Polymerase chain reaction (PCR) amplicons separated on a 3.5% agarose gel showing the distribution of the *sbe1a:gm67* allele among 33 USDA-GEM releases. Reference controls include H99*ae* and GEMS-0067, followed by individual GEM breeding lines and a molecular weight size ladder (LDR; ~400 bp marker indicated). (**A**) Upper gel panel displaying lines 1–17. (**B**) Lower gel panel displaying lines 18–33. Arrowheads identify the 427 bp fragment associated with the *sbe1a:gm67* allele. The allele was detected in GEMS-0067 but was absent from the surveyed GEM releases, indicating that it occurs at low frequency within the evaluated germplasm. Numerical keys correspond to pedigrees listed in Supplemental Table S1.

To evaluate the allele frequency of the Primer I marker, a screening panel was performed across 33 elite USDA Germplasm Enhancement of Maize (GEM) releases (Figure 4). The larger 427 bp amplicon associated with GEMS-0067 was absent from all 33 evaluated GEM releases. All 33 lines exhibited monomorphic migration patterns matching the 419 bp wild-type band of H99*ae*. The 427 bp allele was observed only in GEMS-0067 and Guatemala 209. Notably, two lines sharing ancestry with Guatemala 209—GEMN-0083 and GEMS-0180—did not carry the larger 427 bp amplicon and exhibited the standard wild-type fragment instead.

### 2.3. Evaluation of Cleaved Amplified Polymorphic Sequence (CAPS) Assays

**Figure 5:**
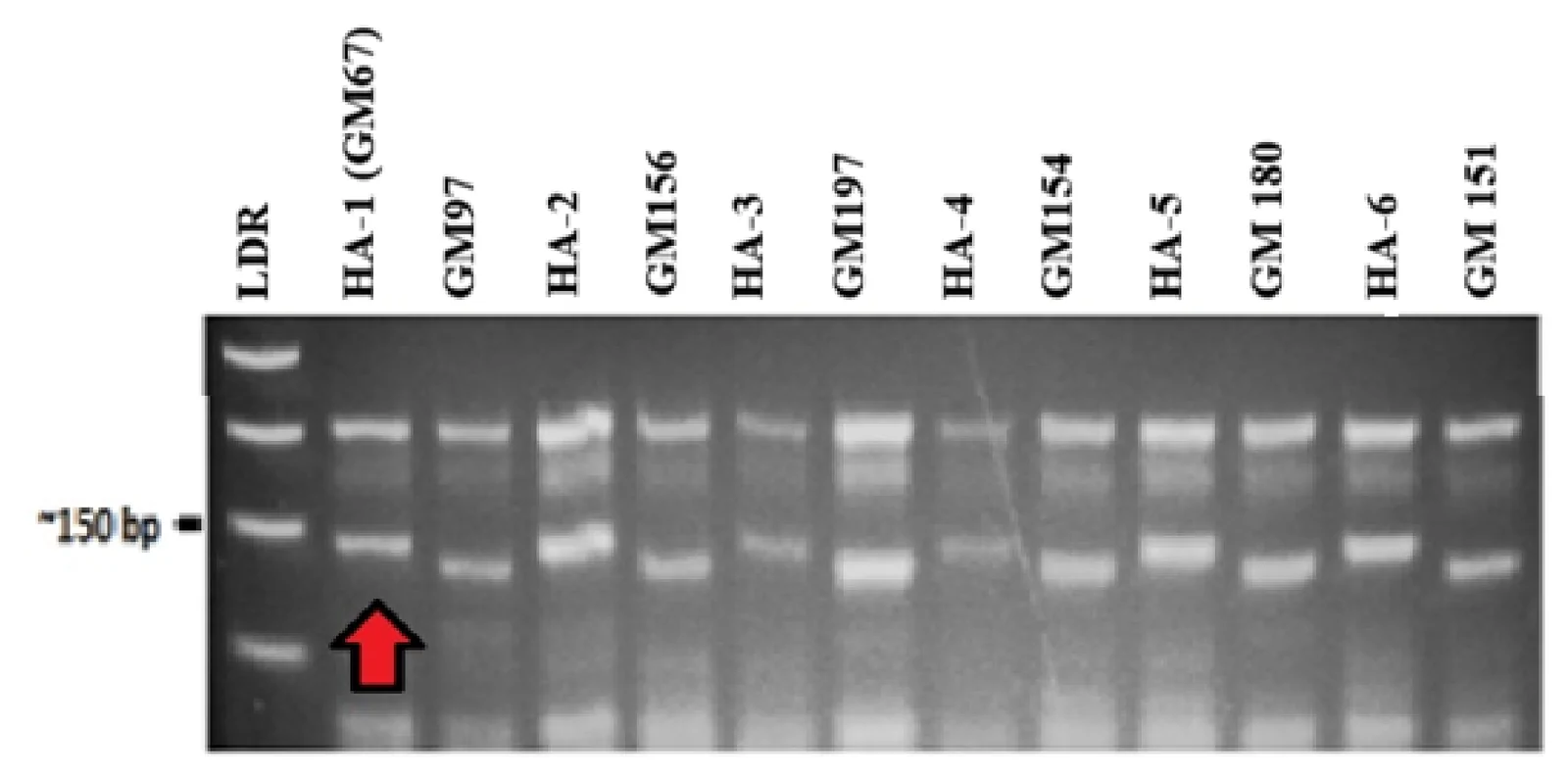
RsaI CAPS marker validation across elite lines and high-amylose derivative families. Electrophoretic separation (2.5% agarose gel, 1 h run) of Primer I amplicons following digestion with RsaI. Lanes are arranged in an alternating pattern to compare normal-endosperm recurrent parents with high-amylose derivative lines. Digestion of the wild-type *Sbe1a* allele yields fragments of 204, 136, and 79 bp, whereas the *sbe1a:gm67* allele produces fragments of 204, 144, and 79 bp. The red arrowhead identifies the diagnostic 144 bp fragment consistently associated with high-amylose derivative lineages. The molecular weight size ladder (LDR) includes a 150 bp reference marker. Lane assignments are provided in Supplemental Table S4.

Direct electrophoretic separation of the uncut 8 bp insertion polymorphism required a 4 h migration on 3.5% Super Fine Resolution (SFR) agarose gels. To shorten this processing window, an RsaI Cleaved Amplified Polymorphic Sequence (CAPS) system was designed. Digestion of the Primer I amplicon with RsaI produced distinct restriction fragment pattern profiles. The wild-type *Sbe1a* allele generated fragments at 204, 136, and 79 bp, whereas the GEMS-0067-derived lines produced fragments at 204, 144, and 79 bp, effectively isolating the 8 bp shift into a single diagnostic fragment (Figure 5, Supplemental Table S3). Alternating gel lane analysis confirmed that high-amylose derivative breeding families (e.g., GEMS-0086 × GEMS-0067 and GEMN-0154 × GEMS-0067) consistently retained the larger 144 bp diagnostic RsaI fragment (Figure 5).

**Figure 6:**
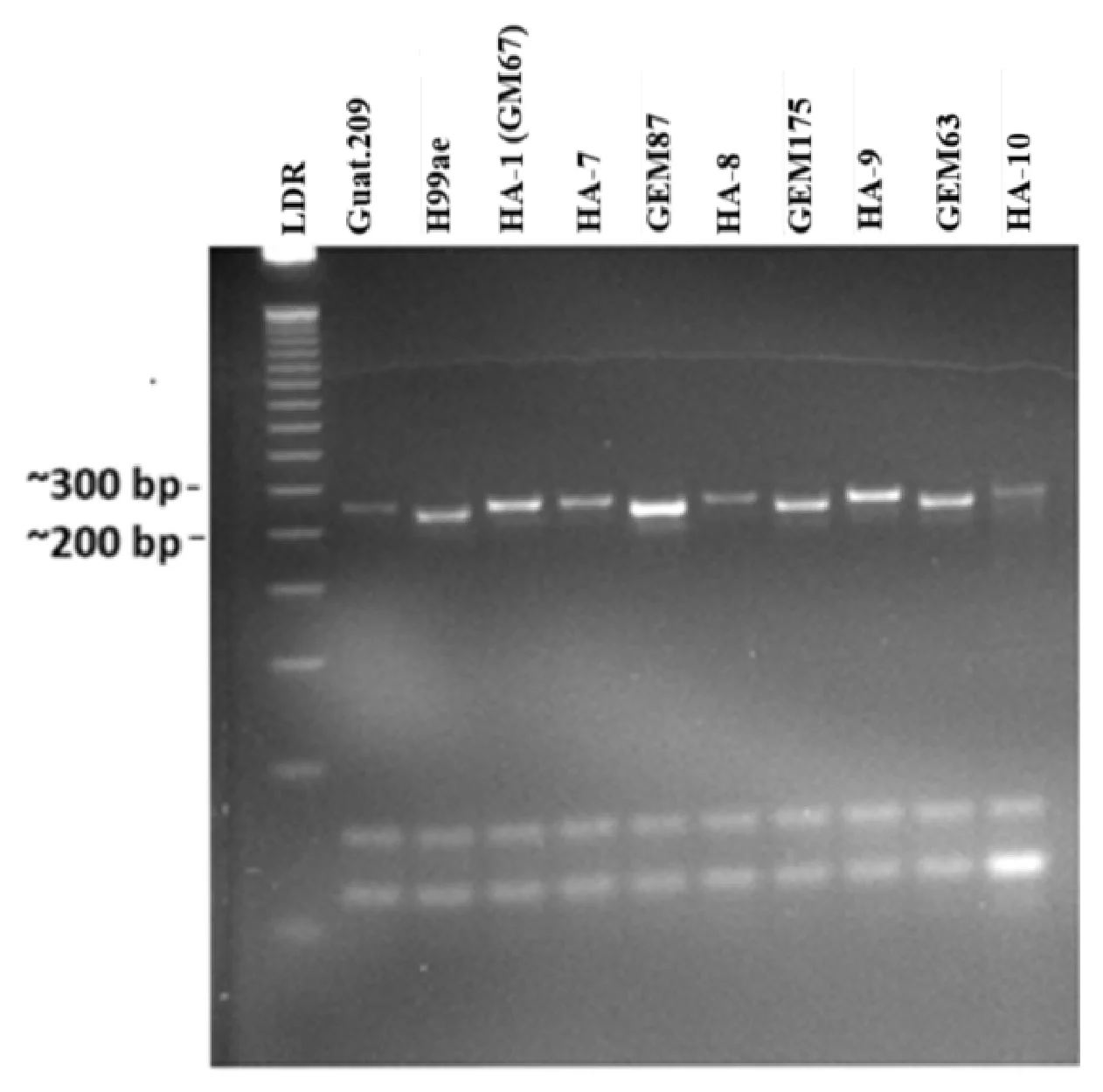
Cleavage tracking of the redesigned, shortened *sbe1a:gm67* marker system following RsaI digestion. Agarose gel separation (3.0% SFR agarose, 3 h run, 120 V) demonstrating resolution of the shortened *sbe1a:gm67* amplicon system (271 bp wild-type versus 279 bp *sbe1a:gm67* allele). Following RsaI digestion, the wild-type allele generates fragments of 136, 79, and 56 bp, whereas the *sbe1a:gm67* allele produces fragments of 144, 79, and 56 bp. Shortening the amplicon allows the polymorphic 144 bp fragment to migrate as the largest fragment, improving ease of visual scoring. Alternating lanes compare normal-endosperm parents with high-amylose families. Lane assignments and sample abbreviations are provided in Supplemental Table S5.

To further simplify the electrophoretic setup, a redesigned, shortened *sbe1a:gm67* marker was engineered, lowering the core size range from 419–427 bp down to 271–279 bp (Figure 1a). Digestion of this shortened amplicon with RsaI yielded predictable fragment matrices: 136, 79, and 56 bp for wild-type lines versus 144, 79, and 56 bp for lines containing the high-amylose modifier allele (Figure 6).

### 2.4. Detection of Heterozygous Individuals

**Figure 7:**
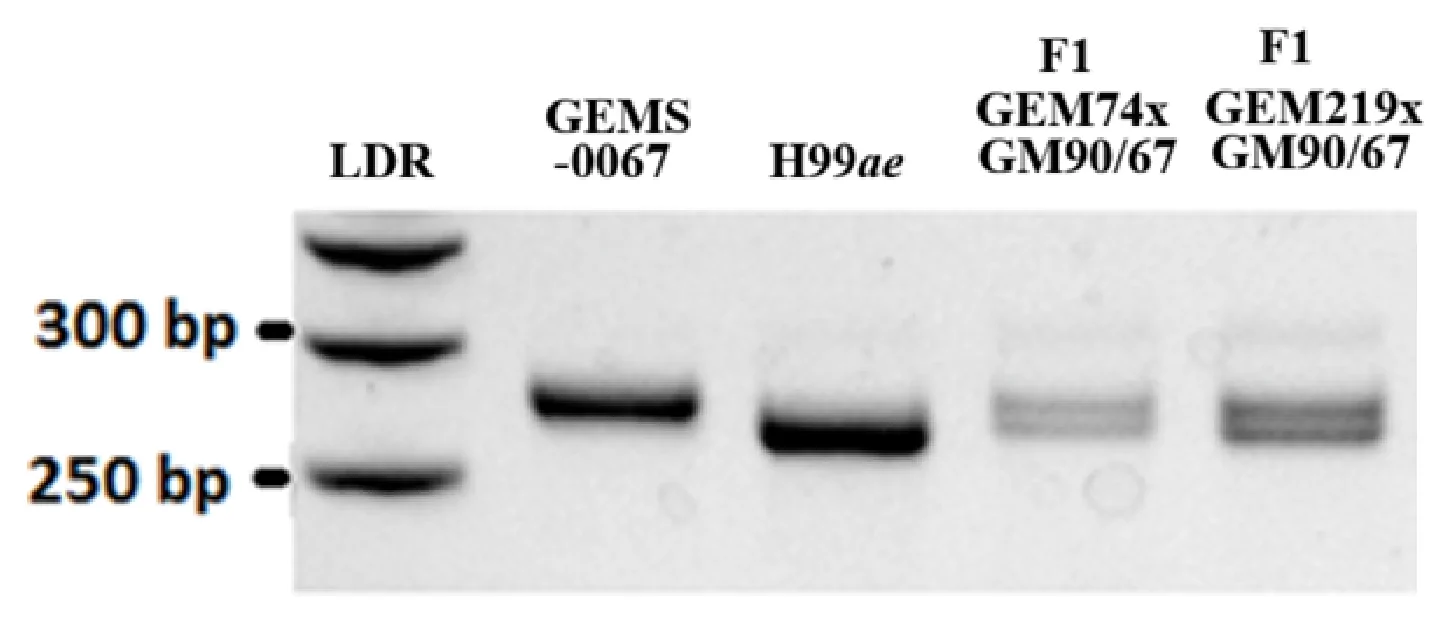
Direct codominant tracking using undigested intact Primer I amplicons. PCR amplification profiles resolved on 3.5% Super Fine Resolution (SFR) agarose gels following a 4 h electrophoresis run at 120 V. Control lanes include homozygous GEMS-0067 and homozygous H99*ae*. F_1_ individuals derived from crosses between high-amylose and normal-endosperm lines display both the 419 bp wild-type and 427 bp *sbe1a:gm67* amplicons simultaneously, demonstrating codominant detection of heterozygous individuals. Sample identities are provided in Supplemental Table S6.

Testing the original intact Primer I system on heterozygous F_1_ individuals derived from crosses between high-amylose and normal-endosperm parental lines successfully yielded codominant dual bands at 419 bp and 427 bp simultaneously (Figure 7). However, standard resolution limits compromised clear discrimination between the bands.

**Figure 8:**
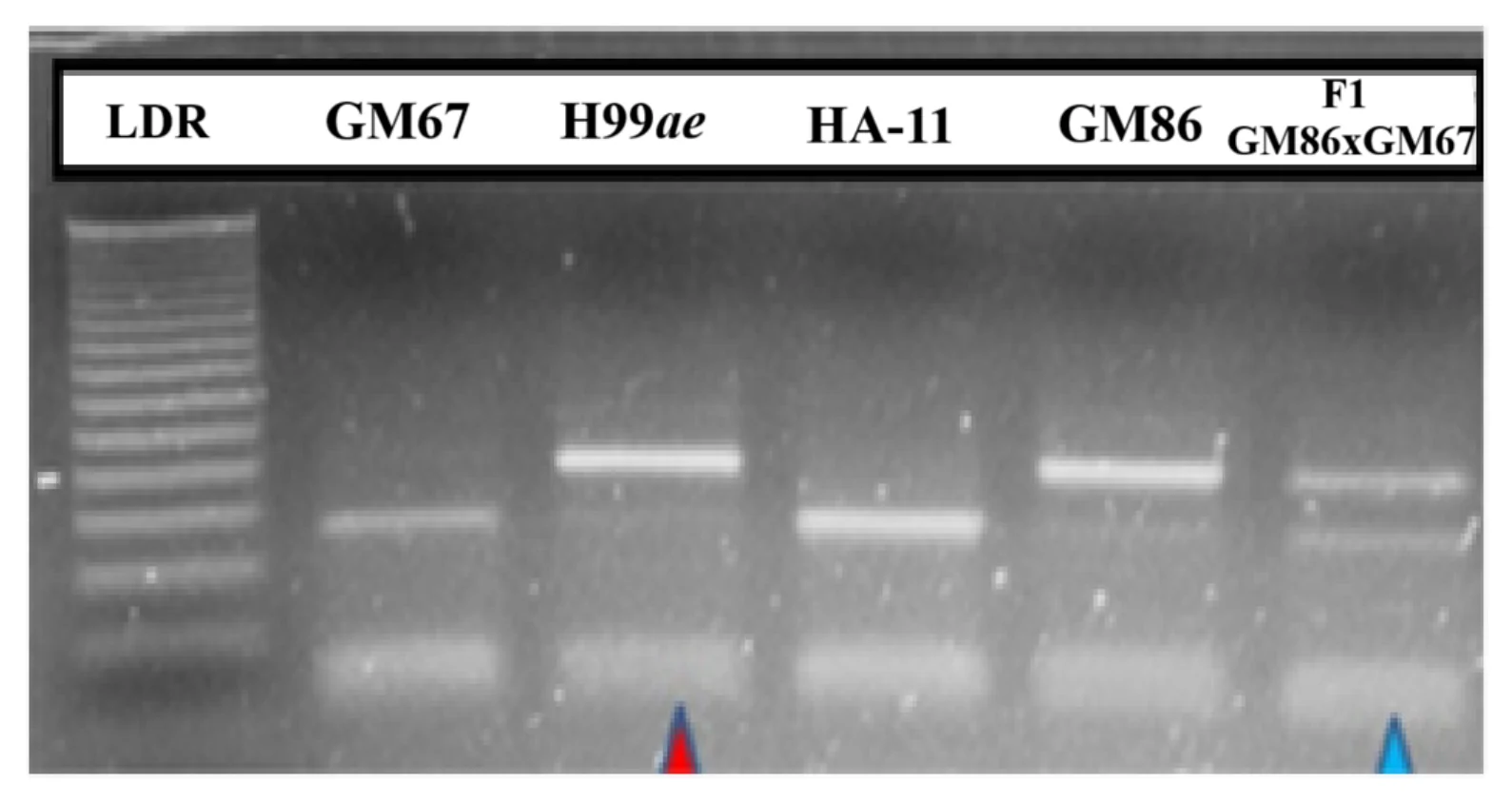
High-resolution codominant mapping utilizing the *sbe1a:gm67*-MslI CAPS assay. Electrophoretic separation (1.0% SFR agarose, 1 h run, 120 V) of amplicons digested with MslI. The intronic TACATA insertion creates an additional MslI recognition site, producing a 182 bp diagnostic fragment in the *sbe1a:gm67* allele relative to the 247 bp wild-type fragment. The red arrowhead indicates the intact 247 bp wild-type fragment present in homozygous H99*ae* (Lane 3). The blue arrowhead identifies a heterozygous F_1_ seedling (Lane 6), which displays both diagnostic fragment lengths simultaneously, demonstrating clear codominant scoring.

Sequence analysis revealed that the 6 bp intronic insertion created an additional cleavage site for the MslI enzyme inside the modifier allele (Figure 2a). Digestion with MslI cut the 419 bp wild-type fragment into 395 bp and 24 bp fragments. In contrast, the 427 bp *sbe1a:gm67* fragment cleaved into 330 bp, 73 bp, and 24 bp fragments (Figure 2b, Supplemental Table S3). When applied to the shortened *sbe1a:gm67* amplicon, MslI digestion converted the minor 8 bp insertion into a distinct codominant size variation, separating a 247 bp wild-type band from a 182 bp mutation band. Heterozygous F_1_ individuals simultaneously displayed both the 247 bp and 182 bp diagnostic bands within a 1 h run on a 1.0% SFR agarose gel (Figure 8).

### 2.5. Native PAGE Zymogram Profiles of Branching Enzymes

**Figure 9:**
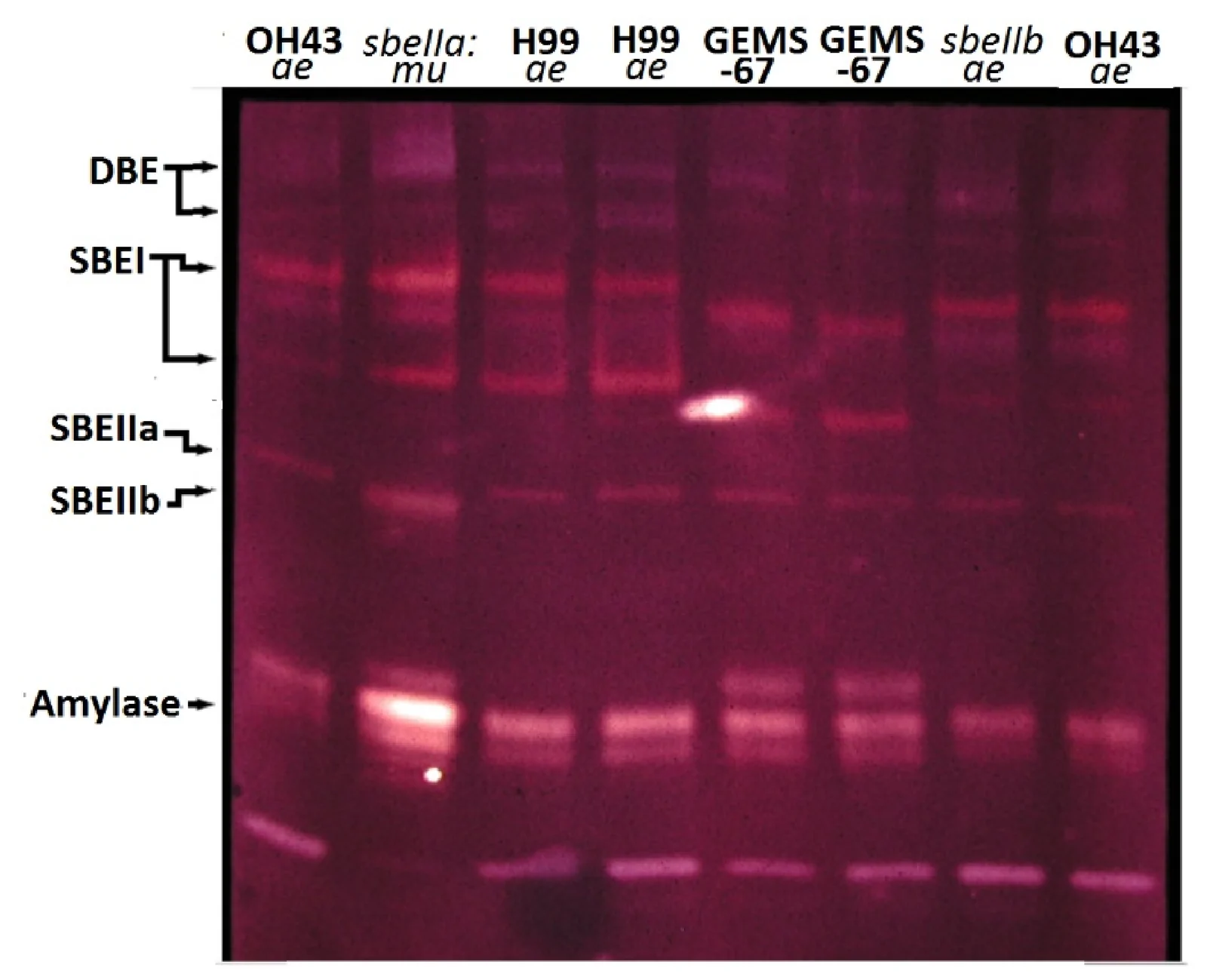
Native PAGE zymogram analysis of starch-metabolizing enzymes in developing maize endosperm. Branching enzyme protein fractions extracted 10 days post-pollination were separated on non-denaturing polyacrylamide gels and visualized by iodine starch staining. Lanes are defined as: 1, Oh43*ae*; 2, *sbe2a::Mu* mutant stock; 3–4, H99*ae* duplicates; 5–6, GEMS-0067 duplicates; 7, *sbe2b* (*ae*) reference stock; and 8, Oh43*ae*. Relative positions for debranching enzymes (DBE), SBEIIa, SBEIIb, and amylases are indicated. An enzyme activity band in the putative SBEI region migrated farther from the well origin in GEMS-0067 than in the reference genotypes. The altered migration pattern was consistently observed in duplicate GEMS-0067 samples.

Native polyacrylamide gel electrophoresis (PAGE) zymogram testing evaluated migration patterns of starch-metabolizing enzyme activity bands from 10-day-old developing kernels (Figure 9). Migration coordinates corresponding to debranching enzymes (DBE), SBEIIa, and amylases were similar across all tested genotypes. Likewise, the activity band corresponding to the SBEIIb region exhibited similar migration in GEMS-0067, Oh43*ae*, and H99*ae*. In contrast, an activity band in the putative SBEI region of GEMS-0067 migrated farther from the well origin, resolving at approximately 2.5 cm and 3.5 cm compared with approximately 2.2 cm and 3.2 cm in the reference genotypes (Figure 9).

## 3. Discussion

### 3.1. Association of the Intron 8 Polymorphism with the High-Amylose Trait

The identification of a quantitative trait locus (QTL) on the short arm of chromosome 5 (5S) implicated the Sbe1a region as a primary candidate modifier gene capable of boosting total starch amylose content from ~55% up to more than 70% when combined with a background homozygous for the *ae* mutation. Prior diagnostic tools for this background focused primarily on exonic Single Nucleotide Polymorphisms (SNPs) within coding domains. Our targeted amplicon scanning approach expanded this focus into non-coding regions, confirming that an 8 bp variation containing a 6 bp TACATA insertion within intron 8 paired with a downstream 2 bp GA insertion is uniquely linked to the high-amylose phenotype of GEMS-0067. Because the materials evaluated in the present study were advanced breeding lines selected over multiple generations for elevated amylose content, the current data establish marker association with the GEMS-0067 modifier background but do not provide an independent assessment of marker-trait segregation within a mapping population. As emphasized in recent discussions of maize QTL mapping, distinguishing tightly linked marker haplotypes from causal variants remains a major challenge for complex traits [10].

### 3.2. Resolution of the Gyawali Sequence Discrepancy

A comparison between our Sanger sequencing dataset and the *Sbe1a* phylogenetic profiles published by Gyawali reveals a noteworthy discrepancy. Gyawali reported the identical TACATA intron 8 insertion in the public line NC350, but noted its absence in his analyzed sample of GEMS-0067 [9]. Given that both GEMS-0067 and NC350 share highly conserved sequence architecture and cluster tightly within the same phylogenetic clade (Clade IV), the apparent absence of this insertion in Gyawali’s GEMS-0067 material warrants careful consideration.

This variation is most likely explained by residual genetic heterogeneity within the historical Guatemala 209 donor gene pool. Because GEMS-0067 was selected out of a segregating, non-fixed tropical population cross (GUAT209:S13), distinct sibling sub-lines may have been distributed during early seed increase cycles. These results are therefore strongly consistent with transmission of the GEMS-0067-associated haplotype from Guatemala 209 into GEMS-0067 and subsequently into derived high-amylose breeding lines. Although both the present study and Gyawali (2016) [9] utilized genomic PCR amplification followed by Sanger sequencing, the contrasting observations concerning the TACATA insertion remain unresolved. Because the original GEMS-0067 materials and chromatogram files were unavailable for reanalysis, residual heterogeneity within historical GEMS-0067 stocks, segregation of chromosomal haplotypes surrounding *Sbe1a*, and divergence among subsequently maintained seed stocks remain plausible explanations for the observed discrepancy. Differences in the underlying sequence resources, particularly for non-coding regions, may have contributed to variation in the representation of intronic polymorphisms. This explanation is supported by our screening data across 33 USDA-GEM releases, where lines with overlapping Guatemala 209 backgrounds (GEMN-0083 and GEMS-0180) did not carry the intron 8 insertion. This indicates that the *sbe1a:gm67* allele was actively segregating and was not fixed during the initial breeding selection stages. Alternatively, variations in PCR primer boundaries or minor structural processing errors across historical draft assemblies could explain the differences in detection. Our results show that this insertion is present in our functional GEMS-0067 stocks and is consistently inherited by all derived high-amylose breeding lines.

### 3.3. Mechanistic Implications of the sbe1a:gm67 Allele

Previous studies have demonstrated that the *ae* (*sbe2b*) mutation present in GEMS-0067 substantially alters starch physicochemical properties, including starch granule morphology, thermal behavior, and amylose concentration [2]. However, these effects alone do not fully explain the exceptionally high amylose levels observed in GEMS-0067, which routinely exceed those expected for *ae* mutants alone. The present study therefore focused on additional modifier variation associated with the chromosome 5 *Sbe1a* region.

At present, neither the intron 8 polymorphism identified in this study nor the previously reported *Sbe1a* polymorphisms described by Chen et al. can be considered functional markers in the strict sense, because a direct causal relationship between these DNA variants and altered SBEI function has not yet been demonstrated [7]. Rather, these markers should currently be viewed as gene-based markers that are strongly associated with the high-amylose phenotype.

The 6 bp TACATA insertion and adjacent 2 bp GA insertion identified within intron 8 of the GEMS-0067 allele represent a candidate polymorphism associated with the chromosome 5 modifier effect; however, a direct functional role has not yet been demonstrated. However, several mechanistic explanations remain possible. First, the intron 8 insertion may alter pre-mRNA processing through effects on splice-site recognition or intronic regulatory sequences, potentially leading to alternative splicing or altered transcript structure. Second, the insertion may simply be linked to another causal polymorphism elsewhere within *Sbe1a* or a nearby regulatory region. Third, the observed differences in native PAGE migration patterns may reflect altered assembly of multienzyme starch biosynthetic complexes rather than alterations in the SBEI polypeptide itself.

This latter possibility warrants consideration because several starch biosynthetic enzymes in developing maize endosperm are known to assemble into multi-subunit protein complexes involving starch synthases and branching enzymes [11]. Altered incorporation of SBEI into such complexes could modify electrophoretic mobility in native PAGE analyses without requiring a change in the molecular weight of the SBEI polypeptide.

Recent reviews have emphasized the complexity of starch biosynthetic regulation in maize endosperm. Zhou et al. [10] highlighted the challenges of identifying causal polymorphisms within complex QTL regions, whereas Zhu et al. [12] summarized evidence that regulatory and non-coding alleles can contribute substantially to phenotypic variation in maize and other crop species. These observations support the possibility that the chromosome 5 *Sbe1a* modifier region functions within a broader regulatory network rather than acting solely through an isolated structural alteration of SBEI.

The native PAGE zymogram results reported here demonstrate that a starch-metabolizing enzyme activity band present in extracts from GEMS-0067 displays altered migration relative to reference lines. However, these data alone do not distinguish between changes in protein size, post-translational modification, or protein-complex formation. Consequently, the results should be considered consistent with, but not proof of, alternative splicing, structural alteration of SBEI, or altered assembly of starch biosynthetic protein complexes.

The native PAGE observations reported here were derived from duplicate samples and are therefore presented as qualitative evidence of altered migration. Quantitative assessment of migration distances across multiple biological replicates was not part of the original study design and will require future investigation.

Further studies will be required to determine the molecular basis of this association. These should include transcript analysis by RT-PCR and cDNA sequencing to evaluate alternative splicing, together with SDS-PAGE and immunoblot analyses to determine whether the SBEI polypeptide itself differs in size from that present in standard alleles. Such experiments would help distinguish between altered protein structure, altered pre-mRNA processing, and altered protein-complex assembly as explanations for the observed native PAGE mobility differences.

### 3.4. Applications for Marker-Assisted Selection (MAS)

From a practical breeding perspective, the codominant MslI CAPS marker developed here represents a practical improvement over traditional visual and chemical screening methods. It is important to note that the marker described here was developed within germplasm fixed for the recessive amylose-extender mutation (*ae/sbe2b*). The *sbe1a:gm67* marker should therefore be viewed as a tool for tracking the GEMS-0067 modifier background rather than as a stand-alone predictor of elevated amylose content. In practical breeding applications, selection for the ae mutation remains essential, whereas the *sbe1a:gm67* marker may facilitate identification and introgression of chromosome 5 modifier haplotypes associated with the high-amylose GEMS-0067 background. Phenotypic selection for high-amylose variants requires waiting until kernel maturity to perform labor-intensive iodine colorimetric assays. Furthermore, identifying heterozygous carriers in early generations is impossible through visual observation alone due to the recessive nature of the *ae* background.

By converting a subtle 8 bp insertion into a distinct restriction pattern difference, the *sbe1a:gm67*-MslI CAPS assay allows rapid, unambiguous genotyping of homozygous and heterozygous individuals at the seedling stage. Implementing this assay can streamline breeding workflows, allowing researchers to introgress the high-amylose modifier trait into elite sweet corn and field corn lines with greater efficiency. Although contemporary breeding programs increasingly integrate genomic prediction, digital phenotyping, and artificial intelligence-based decision tools, accurate low-cost molecular markers remain essential components of these pipelines for validation and selection of favorable haplotypes [13].

### 3.5. Broader Context and Future Directions

Since the registration of GEMS-0067, numerous studies have documented unusual starch properties associated with this germplasm, including elevated resistant starch, altered starch granule morphology, changes in starch digestibility, and exceptionally high apparent amylose levels relative to conventional *ae* materials [3,4,14,15,16,17,18]. These observations suggested the existence of additional modifier loci influencing starch structure and ultimately motivated subsequent genetic investigations of the chromosome 5 modifier region identified by Wu et al. [5,6].

The present study extends this body of work through identification of a candidate intronic polymorphism within *Sbe1a*, development of practical PCR-CAPS markers, and documentation of an altered native PAGE migration pattern associated with GEMS-0067-derived germplasm. Future studies utilizing segregating populations, transcript analysis, protein characterization, and starch structural analyses will be required to determine whether the TACATA insertion represents a causal variant, a linked marker, or a component of a broader chromosome 5 haplotype associated with the high-amylose modifier background.

The novelty of the present study lies not in remapping the chromosome 5 modifier locus, but in extending previous investigations through characterization of a previously undescribed intronic polymorphism within *Sbe1a*, development of practical PCR-CAPS markers suitable for breeding applications, and identification of a distinct native PAGE migration pattern associated with GEMS-0067-derived germplasm. Earlier studies established the chromosome 5 modifier effect and documented coding-region variation within *Sbe1a*; however, a breeder-friendly marker targeting non-coding variation and its association with altered zymogram behavior had not previously been reported.

## 4. Materials and Methods

### 4.1. Germplasm and High-Amylose Line Development

Seeds of GEMS-0067, H99*ae*, and 33 partially inbred Germplasm Enhancement of Maize (GEM) lines representing the stiff-stalk (GEMS) and non-stiff-stalk (GEMN) heterotic groups were obtained from internal breeding stocks or the USDA-GEM program through the North Central Regional Plant Introduction Station, Ames, Iowa (Supplemental Table S1). The open-pollinated tropical maize accession Guatemala 209 (PI 498583) was included as the hypothesized ancestral source of the high-amylose modifier allele.

Amylomaize VII-type high-amylose breeding lines were generated by utilizing GEMS-0067 as the donor parent for both the recessive amylose-extender mutant allele (ae) and the modifier allele *sbe1a*:gm67. Crosses between GEMS-0067 and recipient GEM parent lines were advanced through sequential cycles of self-pollination and selection. Initial selection focused on recovery of the *ae* genotype based on the characteristic wrinkled/tarnished endosperm phenotype. During subsequent generations (F_3_–F_6_), starch was extracted independently from individual ears of each family and evaluated for elevated apparent amylose content. Amylose determinations were performed using duplicate colorimetric analyses for each starch preparation. Commercial Amylomaize V (~55% amylose) and Amylomaize VII (~70% amylose) starches provided by Ingredion Inc. (Westchester, Illinois, USA) were included as internal standards in each analytical batch. Selection decisions were based on repeated evaluations across multiple ears and generations, and only families consistently exhibiting apparent amylose values of approximately 70% or greater were retained for further self-pollination and line advancement. Consequently, the materials included in the present study represent advanced inbred selections subjected to at least four generations of selection for elevated amylose content. In pedigree designations, a single slash (/) indicates an initial cross between a recipient GEM line and the donor line GEMS-0067, whereas a double slash (//) indicates a subsequent cross in which the previously derived high-amylose line served as the donor parent for introgression into an additional recipient background.

### 4.2. Quantitative Colorimetric Amylose Determination

Starch amylose content was quantified across segregating generations using a modified version of the iodine colorimetric procedure described by Williams et al. [19]. Oven-dried starch samples (27.1 mg, dried at 100 °C for 72 h) were placed into 16 mm glass test tubes, mixed with 5 mL of deionized water and one drop of Triton X-100 (LabChem, Inc ., Zelienople, PA, USA), and incubated in a heated sonicating water bath for 30 min. Samples were vortexed, treated with 5 mL of 2 M NaOH, and incubated overnight to achieve complete starch gelatinization.

For colorimetric development, a 100 µL aliquot of the gelatinized starch solution was combined with 5 mL of deionized water, 0.2 mL of 0.5 M HCl, 0.2 mL of iodine reagent (0.2% I_2_ and 2.0% KI), and 5.5 mL of distilled water. Following vortexing and a 30 min incubation, absorbance was recorded at 600 nm utilizing a Beckman DU-520 spectrophotometer (Beckman Instruments, Fullerton, CA, USA). Percent amylose was calculated using a standard regression curve derived from validated commercial maize starch standards containing ~25%, ~55%, and ~70% amylose (Ingredion Inc.).

### 4.3. Primer Set Design and Tiling Strategy

A genomic tiling array consisting of 23 gene-specific primer pairs (designated A–V) was engineered to scan the *Sbe1a* locus for insertion/deletion (indel) polymorphisms associated with the high-amylose modifier effect (Supplemental Table S2). Primers were designed using the NCBI Primer-BLAST software (web version, https://www.ncbi.nlm.nih.gov/tools/primer-blast/, accessed 30 September 2012) against the historical B73 genomic assembly available via MaizeGDB. Standard parameters were set to target primer lengths of 18–25 nucleotides, a GC content of 40–60%, optimal melting synchronization, and minimal secondary primer-dimer structure.

PCR product sizes generated by the primer set ranged from approximately 100 to 500 bp (Supplemental Table S2). Amplification profiles were empirically validated using a panel of controls (GEMS-0067, H99*ae*, Guatemala 209, and GEMN-0087) before large-scale implementation. Primer locations were subsequently compared with the Zm-B73-REFERENCE-NAM-5.0 assembly to facilitate visualization of marker distribution across the contemporary *Sbe1a* gene model (Figure 1a).

### 4.4. DNA Extraction, PCR Amplification, and Gel Electrophoresis

Genomic DNA was isolated from two-week-old greenhouse-grown lyophilized leaf tissues via an SDS-based extraction protocol followed by an RNase treatment step. Isolated DNA concentration and purity profiles were verified via a NanoDrop spectrophotometer (Thermo Fisher Scientific, Wilmington, DE, USA), and working sheets were adjusted to 9 ng µL^−1^ with nuclease-free water.

Polymerase chain reactions (PCR) were conducted in 20 µL total volumes containing 10 µL of GoTaq^®^ Green Master Mix (Promega, Madison, WI, USA), 1.5 µL each of forward and reverse primers, 3 µL of nuclease-free water, and 4 µL of template genomic DNA. Amplifications were executed via a baseline thermocycling sequence—initial denaturation at 95 °C for 5 min; 35 cycles of 94 °C for 30 s, primer-specific annealing (Supplemental Table S2) for 30 s, and 72 °C for 40 s—completed by a final extension at 72 °C for 4 min. Amplicons and enzymatic digests were loaded onto 1.0% to 3.5% Super Fine Resolution (SFR) agarose gels, stained with ethidium bromide, separated at 120 V for 1 to 4 h, and digitally captured under UV transillumination.

### 4.5. Cleaved Amplified Polymorphic Sequence (CAPS) and Sequencing

Targeted amplicons generated by Primer I or the shortened *sbe1a:gm67* marker were digested using RsaI or MslI endonucleases (New England Biolabs, Ipswich, MA, USA). Each cleavage reaction contained PCR product, 1 µL of enzyme, 2.5 µL of bovine serum albumin (BSA), and 2.5 µL of 10× digestion buffer at a 1× working concentration. Reactions were incubated at 37 °C for a minimum of 1 h before size separation on agarose gels.

For sequence verification, intact PCR products generated by Primer I from GEMS-0067 and related breeding derivatives were isolated and submitted to the Iowa State University DNA Facility (Ames, IA, USA) for automated Sanger sequencing using forward and reverse orientations. Sequence chromatograms were assembled and evaluated, and multiple alignments were mapped against the B73 reference standard using the ClustalW algorithm in BioEdit sequence software (version 7.0.9).

### 4.6. Native PAGE Zymogram Enzyme Analysis

Starch-metabolizing enzymatic fractions were analyzed from developing endosperm tissues harvested 10 days post-pollination from Oh43*ae*, H99*ae*, GEMS-0067, a mutant *sbe2a:Mu* stock, and a reference *sbe2b* (*ae*) stock. Tissues were homogenized in extraction buffer, and soluble protein yields were isolated by centrifugation. Proteins were separated by non-denaturing (native) polyacrylamide gel electrophoresis (PAGE) according to procedures described by Colleoni et al. [20]. Gels were incubated in starch-containing reaction buffer and subsequently stained with iodine solution to visualize enzyme activity bands corresponding to branching enzymes (SBE), debranching enzymes (DBE), and amylases. Relative migration distances were measured from the well origin.

## 5. Conclusions

A unique intron 8 polymorphism consisting of a 6 bp TACATA insertion together with a downstream 2 bp GA insertion was identified within the *Sbe1a* locus of the high-amylose maize line GEMS-0067. The polymorphism was absent from 33 surveyed USDA-GEM releases but present in GEMS-0067 and its ancestral source Guatemala 209. PCR-CAPS markers derived from this polymorphism provide practical tools for marker-assisted selection of the GEMS-0067 modifier background. Native PAGE zymogram analyses further revealed a distinct enzyme activity-band migration pattern associated with the GEMS-0067 genotype, although the molecular basis of this observation remains unresolved.

The present study was not designed as a QTL validation experiment, but rather as a characterization of candidate sequence variation within *Sbe1a* and its utility for marker development within breeding materials derived from GEMS-0067. Because the germplasm examined consisted primarily of advanced, selected high-amylose breeding lines, the results establish association rather than causality between the intron 8 polymorphism and the high-amylose phenotype.

Future studies should evaluate segregation of the sbe1a:gm67 marker in populations fixed for the *ae* mutation but segregating for modifier effects, allowing direct assessment of marker-trait association. As emphasized in recent discussions of QTL dissection in maize, multiple markers within a genomic region may exhibit strong trait associations because they track the same underlying haplotype, even when the causal variant has not yet been resolved [10]. Such situations are common during the progression from initial QTL identification to eventual characterization of the functional polymorphism. Additional transcript-level analyses, including RT-PCR and cDNA sequencing, will be required to determine whether the TACATA insertion alters pre-mRNA processing. Likewise, further protein-level studies, including SDS-PAGE, immunoblotting, and expanded biochemical analyses, will be necessary to determine whether altered protein structure or changes in starch-biosynthetic protein complexes underlie the observed zymogram differences.

Despite these limitations, the marker system reported here provides a useful point of reference for future genetic and biochemical studies of the chromosome 5 modifier region associated with high amylose content in GEMS-0067 and related breeding materials.

## Data Availability

Germplasm information is publicly available through the USDA National Plant Germplasm System (GRIN) and MaizeGDB. All data generated or analyzed during this study are included in this article and its Supplementary Materials.

## Supplementary Materials

The following supporting information can be downloaded at https://www.mdpi.com/article/10.3390/plants15203157/s1: Supplemental Table S1: Genetic materials included in this study, including GEMS-0067, its parental sources (Guatemala 209 and H99*ae*), and USDA-GEM germplasm lines screened using the Primer I marker; Supplemental Table S2: Gene-specific primer pairs (A-V) designed to survey the *Sbe1a* locus. PCR product sizes and annealing temperatures correspond to the original primer design and experimental screening conducted during marker development; Supplemental Table S3: Expected PCR and CAPS-RFLP fragment patterns for wild-type, *sbe1a:gm67*, and heterozygous genotypes; Supplemental Table S4: Lane assignments and abbreviated sample codes used in Figure 5; Supplemental Table S5: Lane assignments and abbreviated sample codes used in Figure 6; Supplemental Table S6: Lane assignments and abbreviated sample codes used in Figure 7; Supplemental Table S7: Lane assignments and abbreviated sample codes used in Figure 8.

## Abbreviations

The following abbreviations are used in this manuscript:

CAPS	Cleaved Amplified Polymorphic Sequence
USDA-GEM	United States Department of Agriculture Germplasm Enhancement of Maize Program
