# Supplementary material for: The High-Amylose Maize Line GEMS-0067 Is Associated with an Intron 8 Insertion in Sbe1a, Altered Native PAGE Zymogram Patterns, and Development of PCR Markers for Breeding Applications
Source: Plants (Basel). 2026 Oct 15;15(20):3157. doi: 10.3390/plants15203157 (PMC13709310; doi:10.3390/plants15203157)
Supplement: Supplementary file 1 [file plants-15-03157-s001.zip › plants-4601174-supplementary.pdf]

Supplementary Materials

Supplemental Table S1. Genetic materials included in this study, including GEMS-0067, its parental sources (Guatemala 209 and H99*ae*), and USDA-GEM germplasm lines screened using the Primer I marker.

Pedigree	Lane Code Fig 4	GEM	PI	*ae* status[1]	*sbe1a:gm67* status[2]
Parent sources of GEMS-0067					
Guatemala 209		N/A	PI 498583	absent	present
H99*ae*		N/A	N/A	present	absent
Guat209:S13 x (OH43*ae* x H99*ae*)		GEMS-0067	PI 643420	present	present
GEM recipient lines screened with Primer I					
FS8B(T):N11a-087-001-B	1	GEMN-0097	PI 651534	absent	absent
BR51721:N2012-098-002-B	2	GEMN-0156	PI 658084	absent	absent
DK888:N11(95)-002-021-008-B	3	GEMN-0197	PI 685894	absent	absent
AR13026:N08b15-016-B-B	4	GEMN-0154	N/A	absent	absent
GUAT209:S1308a-047-001-B	5	GEMS-0180[4]	PI 685879	absent	absent
DK212T:N11-040-001-007-B	6	GEMN-0198	PI 685895	absent	absent
MDI022:N2120-284-001-B	7	GEMN-0144	PI 658076	absent	absent
BR52060:S0210-143-001-B	8	GEMS-0142	PI 659679	absent	absent
FS8B(S):S0316-802-001-B	9	GEMS-0079	PI 685818	absent	absent
GUAT209:N1925-081-001-B	10	GEMN-0083[4]	PI 685882	absent	absent
DKXL380N11F2S3_7541-7-B	11	GEMN-0135	PI 685858	absent	absent
PASCO14:N0424-078-001-B	12	GEMN-0190	PI 685887	absent	absent
AR03056:N09-024-001-B	13	GEMN-0087	N/A	absent	absent
BVIR155:S2012-029-001-B	14	GEMS-0175	PI 685874	absent	absent
UR11002:N0308b-086-001-B	15	GEMN-0193	PI 685890	absent	absent
BARBGP2:N08a18-332-001-B	16	GEMN-0110	PI 651537	absent	absent
CH05015:N1204-057-001-B	17	GEMN-0111	PI 651538	absent	absent
UR05017:S0415-180-002-B	18	GEMS-0149	PI 658081	absent	absent
AR17056:N2025-574-001-B	19	GEMN-0174	PI 651545	absent	absent
DKXL212:N11a-139-001-001-B	20	DE4	PI 638552	absent	absent
CHIS740:S1411a-783-002-B	21	GEMN-0191	PI 685888	absent	absent
FS8A(S):S09-362-001-B	22	GEMS-0078	PI 685817	absent	absent
UR11003:S0302-1011-001-B	23	GEMS-0118	PI 651544	absent	absent
ANTIG03:N1218-056-001-B	24	GEMN-0187	PI 685884	absent	absent
NEI9004:S2818-003-001-B	25	GEMS-0146	PI 658078	absent	absent
UR10001:S1813-257-001-B	26	GEMS-0086	PI 685825	absent	absent
CHIS775:N19-042-001-003-B	27	GEMN-0151	PI 685861	absent	absent
UR01089:S0525-519-001-B	28	GEMS-0085	PI 685824	absent	absent
BR51675:N0620-033-001-B	29	GEMN-0140	PI 658073	absent	absent
SCR Gp3:N2017-003-001-B	30	GEMS-0091	PI 651531	absent	absent
SCR Gp3:N14-028-003-003-B	31	GEMN-0137	PI 685860	absent	absent
FS8B(T):N1802-382-001-B	32	GEMN-0081	PI 685820	absent	absent
DREP150:N2011d-624-001-B	33	GEMN-0117	PI 651543	absent	absent

[1]*ae* status: Present indicates evidence for the amylose-extender allele based on pedigree, kernel phenotype, amylose measurements, and/or molecular-marker data. Absent indicates that the *ae* allele is not expected based on pedigree or phenotype.

[2]*Sbe1a:gm67* status: Present indicates detection of the Primer I polymorphism associated with the *sbe1a:gm67* allele. Absent indicates the wild-type allele.

[3]N/A: PI accession number not assigned or not applicable.

[4]GEMN-0083 and GEMS-0180 contain Guatemala 209 ancestry but did not carry the *sbe1a:gm67* allele.

Supplemental Table S2. Gene-specific primer pairs (A-V) designed to survey the *Sbe1a* locus. PCR product sizes and annealing temperatures correspond to the original primer design and experimental screening conducted during marker development.

Primer	Forward Sequence (5′–3′)	Reverse Sequence (5′–3′)	Fragment Size (bp)	Annealing Temp (°C)
A	TTTCGCAGGGTGGCGGCAAT	GGATGGCTGAAGAAAACCAACCACCA	304	56.5
B	GTCCAGTGCAAGGCTCGCCG	GGATGGCTGAAGAAAACCAACCACCA	263	56.5
C	ATAGCACCTGCCCATGCCGC	AGCTCTGCCTCCCTACACCCA	470	57.5
D	GTGAATGGGCACCTGCTGCG	AGCTCTGCCTCCCTACACCCA	353	57.5
E	GGTCTCTTTCTACTGTCCTGGACCAC	GCAGGCTTTGAAGGCCGAGGA	140	55.1
F	GGGATCCTCCTGCTTCTGAAAGGTCTC	GGCTTTGAAGGCCGAGGATGCTT	158	57.2
G	TGGGGTAGACAACACATGGGGCA	ACATGCCAGTGTACATTTCCTTGTCCA	257	55.4
H	CGAGGGATTGCACTCCAAAAGGTTCC	TAGCCATCACCTCCAAGGGCCA	159	56.7
I*	CCGAGTAGCGCTCGTTGCACA	ACAAGGCTCCACTGTCGTCTGC	419	56.7
J	TGCTGCCCCACTGCCCTACT	TCGAGCGCATTCATCGCTTGGT	403	56.7
K	CCAAGCGATGAATGCGCTCGAT	TGAAGCTCGCCTTAATCGGCAAA	196	54.3
L	ACGAGGGGTAAGTCACCAGTTGTA	TCCCAGGCAAATCGCATCCCA	142	54.4
M	ACGAGGGGTAAGTCACCAGTTGTAA	TCCCAGGCAAATCGCATCCCA	142	54.5
N	TGGGATGCGATTTGCCTGGGA	TCGTGGCCAACCTGCGTCAC	225	56.7
O	GCCCTGGACTCTGATGCTCTGGT	TCGTGGCCAACCTGCGTCAC	193	57.9
P	AGTCCTTTCTCCGCCCCGCA	GCCTGGCAGCAGGACGGTTT	103	58.4
Q	CACGTCGCCTGAAGGGGTGC	GCCTGGCAGCAGGACGGTTT	169	58.4
R	CCAGGGGTGCCCGAAACGAA	GCCTGGCAGCAGGACGGTTT	150	57.8
S	CGTCGCCTGAAGGGGTGCC	GCCTGGCAGCAGGACGGTTT	167	58.4
T	CCTTTCTCCGCCCCGCACCT	GCCTGGCAGCAGGACGGTTT	100	58.4
U	GGCACCACACCACTGGGAGC	CTGGAAACGGAGCCGGAGCG	345	58.3
V	CAGCAGGCCGGTCAGCTCAG	CTGGGGGCCATCGACATCGC	486	58.3

*Primer I identified the diagnostic intron 8 polymorphism used for development of the *sbe1a:gm67* marker system.

Supplemental Table S3. Expected PCR and CAPS-RFLP fragment patterns for wild-type, *sbe1a:gm67*, and heterozygous genotypes.

Genotype	Primer I (bp)	Primer I + RsaI (bp)	Primer I + MslI (bp)	Shortened *sbe1a:gm67* (bp)	*sbe1a:gm67* + RsaI (bp)	*sbe1a:gm67* + MslI (bp)
B73 (Wild-Type)	419	204 + 136 + 79	395 + 24	271	136 + 79 + 56	247 + 24
GEMS-0067	427	204 + 144 + 79	330 + 73 + 24	279	144 + 79 + 56	182 + 73 + 24
Heterozygote	427 + 419	204 + 144 + 136 + 79	395 + 330 + 73 + 24	271 + 279	144 + 136 + 79 + 56	247 + 182 + 73 + 24

Supplemental Table S4. Lane assignments and abbreviated sample codes used in Figure 5.

Lane	DNA Sample	Amylose (%)	code
1	Ladder	N/A	LDR
2	GEMS-0067	~70%	HA-1 (GM67)
3	GEMN-0097	~25%	GM97
4	GEMN-0087//GEMN-0111/GEMS-0067	~70%	HA-2
5	GEMN-156	~25%	GM156
6	GEMS-0086/GEMS-0067	~70%	HA-3
7	GEMN-0197	~25%	GM197
8	GEMN-0154/GEMS-0067	~70%	HA-4
9	GEMN- 0154	~25%	GM154
10	GEMN-0087/GEMS-0067	~70%	HA-5
11	GEMS-0180 25%	~25%	GM180
12	GEMN-0099//GEMN-0048/GEMS-0067	~70%	HA-6
13	GEMS-0151	~25%	GM 151

Supplemental Table S5. Lane assignments and abbreviated sample codes used in Figure 6. The validation panel includes reference controls, additional normal-endosperm GEM lines not included in the Primer I survey panel and selected high-amylose breeding lines derived from crosses with GEMS-0067.

Lane	DNA Sample	Amylose (%)	code
1	ladder	N/A	LDR
2	Guatemala209	~25%	Guat.209
3	H99*ae*	~55%	H99*ae*
4	GEMS-0067	~70%	HA-1 (GM67)
5	GEMN-0059/GEMS-0067	~70%	HA-7
6	GEMS-0087	~25%	GEM87
7	GEMN-0059/GEMS-0067	~70%	HA-8
8	GEMS-0175	~25%	GEM175
9	GEMN-0090//GEMN-0048/GEMS-0067	~70%	HA-9
10	GEMS-0063	~25%	GEM63
11	GEMN-0087//GEMN-0048/GEMS-0067	~70%	HA-10

Supplemental Table S6. Lane assignments and abbreviated sample codes used in Figure 7.

Lane	DNA Sample	Amylose (%)	Gel Code
1	ladder	N/A	LDR
2	GEMS-0067	~70%	GM67
3	H99*ae*	~55%	H99*ae*
4	F1 GEMS-0074(female) GEMS90/GEMS67(male)	~25%	F1 GM74xGM90/GM67
5	F1 GEMS-0219 (female) GEMS90/GEMS67(male)	~25%	F1 GM219xGM90/GM67

Supplemental Table S7. Lane assignments and abbreviated sample codes used in Figure 8.

Lane	DNA Sample	Amylose (%)	Gel Code
1	ladder	N/A	LDR
2	GEMS-0067	~70%	GM67
3	H99*ae*	~55%	H99*ae*
4	GEMN-0059 x GEMS-0067	~70%	HA-11
5	GEMS-0086	~25%	GEM86
6	F1 GEMS-0086 (female) GEMS67(male)	~25%	F1 GM86xGM67
